# Cancer and depression in middle-aged and elderly Chinese: The mediating role of sleep quality and the moderating role of social participation diversity – Evidence from CHARLS 2018

**DOI:** 10.1097/MD.0000000000048912

**Published:** 2026-05-15

**Authors:** Yingdan Bai, Zongjing Li, Chen Li, Qian Chen, Yonghui Wan

**Affiliations:** a Renmin Hospital of Wuhan University, Wuhan, P.R. China.

**Keywords:** cancer, CHARLS, depression, sleep quality, social participation diversity

## Abstract

Depression is highly prevalent among individuals with cancer, yet the pathways remain incompletely understood. This study utilized cross-sectional data from the 2018 wave of the China Health and Retirement Longitudinal Study. Cancer was identified from harmonized and health files. Depression was assessed using the 10-item Center for Epidemiologic Studies Depression Scale. Sleep quality was proxied by a single item, which captures one dimension of sleep disturbance. Social participation diversity was constructed from 6 types of activities and summarized as a variety index. Covariates included age, sex, education, partnership, urban residence, retirement, drinking, smoking, and comorbidities. Mediation analysis was conducted using nonparametric bootstrap with 5000 resamples. Moderation was tested via linear models incorporating a sleep quality × social participation interaction term. The analytical sample included 17,550 adults aged 45 years and above. The total effect of cancer on depression was 2.157 (95% confidence interval [CI]: 0.458–3.856). The indirect effect mediated through poor sleep quality was 0.265 (95% CI: 0.028–0.587, *P* = .026), accounting for 12.29% of the total effect, indicating that sleep quality partially statistically mediated the relationship between cancer and depression. The direct effect of cancer on depression was 1.892 (95% CI: 0.246–3.538, *P* = .024). Social participation diversity attenuated the path from sleep to depression (sleep × participation interaction: β = −0.134, standard error = 0.043, *P* = .002). Among Chinese adults aged 45 and older, sleep quality statistically accounted for a modest portion of the cancer–depression association, and social participation diversity modified the sleep–depression association. Given the cross-sectional design and the single-item sleep measure, findings should be interpreted cautiously with respect to temporality and causality. Interventions addressing sleep problems and supporting social engagement warrant further evaluation in longitudinal and interventional studies.

## 1. Introduction

China’s cancer burden has risen rapidly alongside population aging. According to GLOBOCAN 2022 estimates, approximately 4.82 million new cancer cases and 2.57 million cancer deaths occurred in China,^[1]^ accounting for about 24% of the global cancer incidence.^[2]^ The 5-year prevalence of diagnosed cancer in China now exceeds 10 million cases. At the same time, China’s population is aging: by the end of 2023, 296.97 million people (21.1% of the population) were aged ≥ 60.^[3]^ This demographic shift portends a growing number of older cancer patients and survivors, underscoring the need to address their mental health.

Depression is one of the most common, and treatable, psychiatric conditions among cancer patients.^[4]^ It is estimated to affect approximately 20 to 30% of individuals with cancer at any given time, a prevalence several-fold higher than in the general population.^[5]^ By contrast, studies using structured clinical interviews in oncologic settings report point prevalence of major depression ranging from roughly 5 to 16% in outpatients and 4 to 14% in inpatients, to as high as 7 to 49% in palliative care settings.^[6]^ Depression in cancer is associated with poorer adherence to treatment, slower recovery, reduced quality of life, and elevated mortality risk.^[4]^ Meta-analyses confirm that cancer patients with depression have significantly higher all-cause and cancer-specific mortality. For example, a recent review of 65 cohort studies found that depression after cancer diagnosis was associated with a 38% increase in the risk of cancer-related death (pooled hazard ratio [HR] = 1.38). These findings highlight why depression prevention and management is a priority in oncology care.

Sleep problems are highly prevalent in cancer and may play a role in depression etiology. Recent systematic reviews report that approximately 60% of people with cancer experience insomnia or significant sleep disturbance.^[7]^ Such sleep problems often persist beyond active treatment into survivorship: for instance, nearly 40% of cancer survivors report ongoing sleep difficulties up to 5 years after diagnosis, and even at 9 years post-diagnosis over half of survivors have clinically significant insomnia or sleep complaints.^[8]^ There is strong evidence that insomnia and short sleep duration are prospective risk factors for depression in general populations.^[9,10]^ Non-depressed individuals with chronic insomnia have roughly double to triple the odds of developing major depression compared to good sleepers.^[11]^ Proposed mechanisms linking sleep disturbance to depression include upregulation of inflammatory pathways and stress axes and impairments in emotion regulation.^[10]^ Sleep disruption triggers neuroimmune changes, for example, elevated proinflammatory cytokines and HPA-axis activation, which are thought to promote depressive symptoms. Thus, an emerging hypothesis is a chain of influence whereby cancer-related stressors disrupt sleep, and poor sleep in turn contributes to depression.

Beyond these risk pathways, social participation is thought to protect mental health in later life. The classic stress-buffering model posits that social support and participation mitigate the psychological impact of stressors such as serious illness.^[12]^ The concept of social participation encompasses involvement in personal, community, and civic activities that provide interaction with others.^[13,14]^ The breadth (variety of activity types) of participation can be quantified. Greater social integration is associated with numerous positive outcomes in older adults, including better physical and cognitive functioning, lower rates of disability, higher life satisfaction, and reduced mortality. In China’s aging population, analyses of nationally representative cohorts such as China Health and Retirement Longitudinal Study (CHARLS) have linked higher social participation to fewer depressive symptoms, better self-reported health, and longevity.^[15]^ For example, a recent longitudinal study of adults ≥ 65 in China found that those with low social participation had a significantly higher risk of depression over time. Social participation may confer a sense of purpose, social support, and behavioral activation, which help seniors cope with health-related stressors.

However, evidence explicitly modeling sleep quality as a mediator in the cancer–depression relationship and social participation diversity as a moderator remains limited. We leverage data from CHARLS to investigate these psychosocial pathways. We hypothesized that: cancer is associated with more depression; sleep problems partially mediate this association; and social participation moderates the associations (potentially on the a-path and/or the direct c’-path), such that higher participation attenuates the adverse effects of cancer and poor sleep on depression.

## 2. Methods

### 2.1. Data source and study population

The CHARLS, launched by Peking University in 2011, is a nationally representative longitudinal cohort of permanent residents aged 45 and older in China.^[16]^ Using a multistage, stratified, probability-proportional sampling approach, the study systematically collects information on individual health status, socioeconomic status and family structure, healthcare utilization, lifestyle, and social participation. The methodological design of CHARLS aligns with that of the Health and Retirement Study and the Survey of Health, Ageing and Retirement in Europe, ensuring data comparability and representativeness for international comparisons. This study used data from the 2018 follow-up (wave 4) and incorporated bridging variables provided by the Harmonized CHARLS.

To ensure comparability, we first included individuals aged ≥ 45 years from the 2018 survey. We then excluded individuals based on the following criteria: missing information required for a rigorous cancer assessment (physician diagnosis and/or location/treatment clues could not be determined); missing depression total score (CES-D 10); missing sleep quality items; missing key variables for social participation (either core component of activity participation or frequency was missing, resulting in an inability to define a moderating variable).

### 2.2. Variables

#### 2.2.1. Assessment of cancer

Cancer was defined using the interviewer-administered CHARLS item on physician diagnosis. Participants were asked: “Have you ever been diagnosed with cancer or a malignant tumor (excluding minor skin cancers) by a doctor?” (yes/no). Those answering “yes” were coded as cancer = 1 and all others as cancer = 0, constituting the strict definition for primary analyses, consistent with prior CHARLS-based cancer survivorship studies.^[17]^

#### 2.2.2. Assessment of sleep quality

Sleep quality was assessed with the CHARLS item “My sleep was restless in the last week.” Participants responded on a 4-point scale (0 = rarely or none of the time < 1 day; 1 = some of the time 1–2 days; 2 = occasionally or a moderate amount of the time 3–4 days; 3 = most or all of the time 5–7 days). Higher scores indicate worse sleep quality. This operationalization has been commonly used in CHARLS-based studies as a pragmatic indicator of sleep disturbance in large-scale surveys.^[18]^ Nevertheless, sleep quality was measured using a single self-reported item, which captures only one dimension of sleep disturbance and is not equivalent to a validated multi-item sleep scale.

#### 2.2.3. Assessment of depression

Depression were measured with the 10-item Center for Epidemiologic Studies Depression Scale (CES-D 10), a brief screening instrument that sums responses to 10 affective and somatic items to yield a continuous total score (range 0–30; higher scores indicate more severe symptoms). Each item is rated on a 4-point frequency scale referencing the past week; positively worded items are reverse-coded before summation, consistent with Harmonized CHARLS conventions. The CES-D 10 has been extensively used in Chinese middle-aged and older adult populations and has demonstrated good psychometric properties across community samples,^[19,20]^ supporting its use for epidemiologic analyses in this age group.

#### 2.2.4. Assessment of social participation diversity

Social participation diversity was measured from the CHARLS social activity module. Respondents were asked whether, during the past month, they engaged in each of the following activities: visiting or socializing with friends; playing mahjong or other board games; attending sports or social clubs; taking part in community or association activities; volunteering; providing unpaid help to relatives, friends, or neighbors; and using the internet for leisure. In line with prior CHARLS-based work and given the low prevalence of items (5) and (6), these 2 were combined as a single “voluntary activity” domain.^[21]^ Each domain was dichotomized (No/Yes) according to whether any participation was reported in the reference period. Because this count measure was right-skewed with a relatively sparse upper tail, and to avoid small cell sizes that can destabilize interaction estimates, we collapsed the index into 4 categories (0, 1, 2, and ≥ 3 types) for descriptive presentation and interpretability. This categorization has also been used in prior CHARLS-based work and facilitates comparability across studies.^[22]^

#### 2.2.5. Assessment of covariates

We prespecified a set of demographic, socioeconomic, lifestyle, and clinical covariates based on prior evidence of their associations with both cancer and depressive symptoms, and their potential to confound relations involving sleep and social participation. These included age (years, continuous), sex (male/female), educational attainment (categorized as illiterate, primary or below, middle school, high school or above), partnership status (yes/no), place of residence (urban vs rural), retirement (yes/no), current drinking (yes/no), current smoking (yes/no), and doctor-diagnosed dyslipidaemia, diabetes, heart disease, and stroke (each yes/no).

### 2.3. Statistical analysis

Descriptive statistics were used to summarize sample characteristics (categorical variables as n [%]; continuous variables as mean ± SD and range). Group differences by physician-diagnosed cancer (yes/no) were evaluated using χ^2^/Fisher exact tests for categorical variables and Welch t tests for continuous variables; skewness and kurtosis were inspected to screen normality, with Wilcoxon rank-sum tests applied when assumptions were untenable. Because missingness in covariates was limited but non-negligible, we applied multiple imputation by chained equations (multiple imputation by chained equations; m = 20) for covariates only. The primary association was examined with multivariable linear regression of CES-D-10 score on cancer, adjusting a priori for age, sex, education, partnership, urban residence, retirement, drinking, smoking, dyslipidaemia, diabetes, heart disease, and stroke; We used HC3 robust standard errors and inspected standard diagnostics.

To test mechanism, we conducted a statistical mediation analysis treating the sleep quality as mediator. Given the cross-sectional design, this mediation should be interpreted as a statistical decomposition of associations rather than causal mediation. We estimated the total effect, direct effect (ADE), indirect effect (ACME), and proportion mediated using nonparametric bootstrap (5000 resamples).

Effect modification by social participation diversity was tested by adding interaction terms to fully adjusted linear models. Specifically, we fitted a cancer × diversity interaction in the cancer→depression model and a sleep × diversity interaction in the sleep→depression model. For interpretability, continuous predictors entering interactions were mean-centered and standardized (z-scores), and we reported simple slopes at − 1 SD, the mean, and + 1 SD of diversity. All tests were 2-sided with α = 0.05. All analyses were performed in R 4.5.1.

## 3. Results

### 3.1. Participant characteristics

Among 17,550 CHARLS participants aged ≥ 45 years, 383 (2.2%) reported physician-diagnosed cancer. Compared with non-cancer participants, those with cancer were older (mean 63 ± 9 vs 61 ± 10 years; *P* = .001) and more often female (63% vs 52%; *P* < .001). They were more likely to be retired (56% vs 32%; *P* < .001), reported less current drinking (25% vs 35%; *P* < .001) and less current smoking (16% vs 28%; *P* < .001), and had a higher burden of cardiometabolic comorbidity, including dyslipidaemia (32% vs 22%; *P* < .001), diabetes (21% vs 13%; *P* < .001), and heart disease (28% vs 20%; *P* < .001); stroke prevalence did not differ (*P* > .9). Sleep quality was worse in the cancer group (category “4” 29% vs 22%; category “1” 36% vs 46%; *P* < .001), and the CES-D 10 score was higher (10 ± 7 vs 8 ± 6; *P* < .001). Education, marital/partnership status, and urban–rural residence showed small to modest differences (urban 52% vs 60%; *P* < .001). Any social participation did not differ by cancer status (*P* > .9). Detailed counts and percentages are reported in Table 1.

**Table 1:** Baseline characteristics (overall and by cancer status).

	Total sample (N = 17,550)	Non_CA(N = 17,167)	CA(N = 383)	*P* value
	n (%)	n (%)	n (%)	
Age				**.001**
Mean ± SD	61 ± 10	61 ± 10	63 ± 9	
Gender				**<.001**
Male	8393 (48)	8250 (48)	143 (37)	
Female	9157 (52)	8917 (52)	240 (63)	
Education				0.6
Illiterate	3980 (23)	3902 (23)	78 (20)	
≤Primary school	8099 (46)	7920 (46)	179 (47)	
Middle school	3440 (20)	3357 (20)	83 (22)	
≥High school	2031 (12)	1988 (12)	43 (11)	
Marital status				0.3
Single	2395 (14)	2336 (14)	59 (15)	
Partnered	15,155 (86)	14,831 (86)	324 (85)	
Retirement				**<.001**
No	11,776 (67)	11,608 (68)	168 (44)	
Yes	5774 (33)	5559 (32)	215 (56)	
Residential area				**<.001**
Rural	6985 (40)	6800 (40)	185 (48)	
Urban	10,565 (60)	10,367 (60)	198 (52)	
Alcohol consumption				**<.001**
No	11,455 (65)	11,166 (65)	289 (75)	
Yes	6095 (35)	6001 (35)	94 (25)	
Smoking				**<.001**
No	12,756 (73)	12,434 (72)	322 (84)	
Yes	4794 (27)	4733 (28)	61 (16)	
Dyslipidemia				**<.001**
No	13,603 (78)	13,342 (78)	261 (68)	
Yes	3947 (22)	3825 (22)	122 (32)	
Diabetes				**<.001**
No	15,252 (87)	14,951 (87)	301 (79)	
Yes	2298 (13)	2216 (13)	82 (21)	
Heart problems				**<.001**
No	14,052 (80)	13,776 (80)	276 (72)	
Yes	3498 (20)	3391 (20)	107 (28)	
Stroke				>.9
No	16,382 (93)	16,025 (93)	357 (93)	
Yes	1168 (6.7)	1142 (6.7)	26 (6.8)	
Sleep quality				**<.001**
0	8092 (46)	7954 (46)	138 (36)	
1	2867 (16)	2805 (16)	62 (16)	
2	2673 (15)	2602 (15)	71 (19)	
3	3918 (22)	3806 (22)	112 (29)	
Depression				**<.001**
Mean ± SD	8 ± 6	8 ± 6	10 ± 7	
Social participation				>.9
No	6760(55.6)	2342(53.4)	4418(56.8)	
Yes	5405(44.4)	2045(46.6)	3360(43.2)	

### 3.2. Mediation of the relationship between cancer and depression by sleep quality

Table 2 summarizes the statistical mediation of sleep quality in the cancer–depression association. Cancer was associated with poorer sleep (path a: β = 0.418, 95% CI 0.023–0.813; *P* = .038). In turn, poorer sleep was related to higher depression (path b: β = 0.634, 95% CI 0.441–0.827; *P* < .001). The total effect of cancer on depression was β = 2.157 (95% CI 0.458–3.856; *P* = .013), of which β = 1.892 (95% CI 0.246–3.538; *P* = .024) remained after accounting for sleep. The indirect effect was 0.265 (95% CI 0.028–0.587; *P* = .026), corresponding to 12.29% of the total association. Thus, the indirect component was statistically significant but modest in magnitude.

**Table 2:** Mediation of the relationship between cancer and depression by sleep quality.

	Estimate	95% CI lower	95% CI upper	*P* value
Total effect	2.157	0.458	3.856	.013
Direct effect	1.892	0.246	3.538	.024
Mediator path a (Cancer → Sleep)	0.418	0.023	0.813	.038
Mediator path b (Sleep → Depresion)	0.634	0.441	0.827	<.001
Indirect	0.265	0.028	0.587	.026
Proportion mediated	12.29%	-	-	-
Observations	17,550	-	-	-

### 3.3. Moderation by social participation diversity

Table 3 reports the results of the moderating effect of social participation diversity. Restricting effect-modification analyses to social participation diversity, we found consistent buffering of the associations linking cancer and sleep quality to depression. The cancer–depression association was significantly weaker at higher diversity (interaction β=−0.761, 95% CI − 1.454 to − 0.068; *P* = .031). Likewise, the association of poorer sleep (per 1-SD worse sleep) with higher depression was attenuated as diversity increased (interaction β=−0.136, 95% CI − 0.219 to − 0.052; *P* = .001). For cancer→sleep, the interaction was directionally similar but marginal (β=−0.124, 95% CI − 0.249 to 0.001; *P* = .053). These estimates derive from fully adjusted linear models (age, sex, partnership, urban residence, retirement, drinking, smoking, dyslipidaemia, diabetes, heart disease, stroke; n = 17,550) with HC3 robust standard errors.

**Table 3:** Effect modification by social participation diversity (interaction terms).

Outcome	Exposure	Moderator	β_interaction (95% CI)	*P* value
Depression	Cancer	social participation diversity	−0.761 (−1.454, −0.068)	**0.031**
Depression	Sleep quality	social participation diversity	−0.136 (−0.219, −0.052)	**0.001**
Sleep quality	Cancer	social participation diversity	−0.124 (−0.249, 0.001)	0.053

Simple slopes (magnitude and absolute differences). At low diversity (−1 SD), cancer was associated with a larger increase in depression (β = 2.506, 95% CI 1.491–3.521; *P* < .001), which diminished at the mean (β = 1.745, 1.032–2.458; *P* < .001) and was smallest at high diversity (+1 SD) (β = 0.983, 0.010–1.956; *P* = .048). The between-level difference (−1 SD→+1 SD) was − 1.523, implying an ≈ 61% relative attenuation (0.983/2.506 ≈ 0.39). For the sleep→depression pathway, the per-SD effect of worse sleep was 3.530 (95% CI 3.407–3.652; *P* < .001) at − 1 SD, 3.394 (3.307–3.481; *P* < 0·001) at the mean, and 3.259 (3.140–3.378; *P* < .001) at + 1 SD; the cross-level difference was − 0.271 (≈7.7% attenuation; 0.271/3.530). For cancer→sleep, slopes were 0.320 (95% CI 0.141–0.498; *P* < .001), 0.196 (0.072–0.319; *P* = .002), and 0.072 (−0.102–0.245; *P* = .417) at − 1 SD, mean, and + 1 SD, respectively. Overall, while interactions were statistically significant, the absolute contrasts, particularly for sleep→depression, were modest.

**Figure 1.**
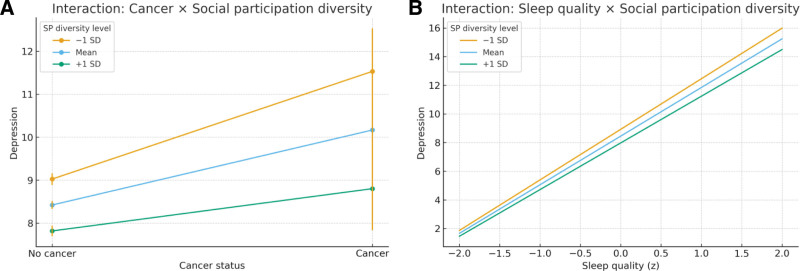
Effect modification by social participation diversity on (A) cancer–depression and (B) sleep–depression associations (fully adjusted).

Figure 1. Panel A plots Depression by cancer status at − 1 SD, mean, and + 1 SD of participation diversity (fully adjusted), showing a visibly smaller cancer–depression contrast at higher diversity. Panel B plots Depression across the sleep z-score with separate lines for diversity levels; the slope is steepest at low diversity and flattest at high diversity, illustrating attenuation of the sleep–depression association.

## 4. Discussion

In this large sample of middle-aged and older Chinese adults, cancer survivors reported significantly more depression than peers without cancer. We also observed that sleep quality statistically accounted for a modest portion of the cancer–depression association: the indirect effect through poorer sleep represented 12.29% of the total association. Social participation diversity significantly moderated both the association between cancer and depression and the association between sleep quality and depression, consistent with a stress-buffering effect. Taken together, these findings integrate sleep-related statistical mediation and social engagement–related effect modification within a single framework, extending prior work on depression among cancer survivors^[23,24]^and on sleep disturbance in relation to depressive symptoms,^[25]^ as well as gerontological evidence on social engagement and mental health in later life.

Importantly, because the data are cross-sectional, the mediation and moderation results should be interpreted as statistical decompositions and effect modification, rather than evidence of temporal or causal mechanisms. Cancer and its treatments can induce inflammation and dysregulation of stress-hormone (HPA) axes, and insomnia itself may exacerbate inflammatory signaling.^[26]^ These factors can adversely affect neurotransmitter function and emotion regulation, increasing susceptibility to depression. At the same time, social participation may counteract these processes by providing emotional support, behavioral activation, structured daily activity, and cognitive stimulation, consistent with the classic stress-buffering model of social support.^[27]^ In our study, participants with greater social participation were less affected mood-wise by poor sleep quality, suggesting that an active social life can psychologically “insulate” individuals against some of the mood-destabilizing effects of sleep disturbance. This observation dovetails with broader evidence that strong social ties and support can enhance resilience in the face of health stressors.

These findings have several practical implications. First, they highlight 2 modifiable targets for improving mental health in cancer survivors: sleep disturbance and social isolation. Insomnia is prevalent in cancer patients yet often undertreated; evidence-based interventions such as cognitive-behavioral therapy for insomnia (CBT-I) have been shown to not only improve sleep but also reduce depression and fatigue in cancer survivors.^[28]^ Addressing sleep problems in survivorship care could therefore yield dual benefits for physical and psychological well-being. Second, our results underscore the protective value of social participation. Encouraging cancer survivors to engage in social activities, through community programs, support groups, or rehabilitation initiatives that incorporate social elements, may buffer the impact of stress and enhance coping. Randomized trials and meta-analyses indicate that psychosocial interventions (e.g., supportive-expressive therapy, structured group activities) can significantly alleviate distress and improve quality of life in cancer patients.^[29]^ Incorporating strategies to maintain or increase social connectedness in survivorship plans is thus recommended. Notably, depression in cancer has been linked to worse disease outcomes including higher mortality,^[30–32]^ so mitigating depressive symptoms is not only important for quality of life but could potentially influence clinical prognosis.^[32]^ Recognizing this, oncology guidelines call for routine distress screening and comprehensive psychosocial care as standard components of cancer management.^[33]^ However, depression and other distress often remain under-recognized and undertreated in practice. Greater efforts are needed to implement these guidelines and integrate mental health services into oncology settings. Initiatives to make distress the “sixth vital sign” in cancer care have been proposed to ensure systematic attention to patients’ psychological needs. Our findings add impetus to such efforts, suggesting that a combination of managing insomnia and fostering social support could reduce the depression burden in this growing population. Moreover, beyond reducing depressive symptoms, maintaining social engagement may confer broader benefits for older cancer survivors, including improved life satisfaction and well-being.^[34]^

Strengths and Limitations: Key strengths of this study include the large, population-based sample, which enhances generalizability to middle-aged and older Chinese adults. The CHARLS data allowed us to examine real-world psychosocial factors in a nationwide context, complementing clinical studies. However, several limitations must be noted. First, the cross-sectional design limits causal inference. We cannot definitively establish the temporal ordering of cancer, sleep disturbance, and depression. It is plausible that depression or poor sleep could influence social participation (e.g., withdrawal) rather than vice versa. Longitudinal analysis is needed in future CHARLS waves to confirm directionality. Second, sleep quality was assessed using a single self-reported item (“my sleep was restless”). Although this item is commonly used as an indicator of sleep disturbance in large-scale surveys, it does not capture the multidimensional nature of sleep and is not equivalent to a validated multi-item sleep instrument. This is relevant because sleep was modeled as the mediator and also entered the moderation pathway; therefore, limited measurement precision may introduce uncertainty in both the estimated indirect effect and the sleep × social participation diversity interaction. In general, non-differential measurement error tends to attenuate associations, suggesting that our estimates may be conservative. Future studies incorporating validated multi-item sleep scales (e.g., insomnia symptom measures) and, where feasible, objective assessments (e.g., actigraphy) would help strengthen inference. Third, all data were self-reported. Depression was assessed by CES-D rather than clinical diagnosis; cancer was self-reported (though CHARLS does attempt to verify with medical records when available). Self-report can introduce bias or error (e.g. underreporting of depression due to stigma). That said, CES-D is a validated scale widely used in epidemiology, and self-reports are often the only feasible way to gauge psychosocial variables in large surveys. Fourth, there may be residual confounding by unmeasured factors. For instance, personality traits (e.g. extroversion) might influence both social participation and depression risk. Lastly, the CHARLS cancer sample included all cancer types and stages; we could not stratify by cancer type or time since diagnosis. Depression and sleep issues can vary by cancer site (e.g. higher in lung vs skin cancer) and phase (active treatment vs long-term survivorship). Our results represent an average effect across a heterogeneous survivor group. Future work with larger cancer subsamples should explore differences by cancer type, treatment status, or severity.

Despite these limitations, our study provides novel evidence from a non-Western aging population to inform the psychosocial oncology literature. It underscores that sleep problems mediate a modest share of the cancer–depression link, and social participation diversity can mitigate the impact of both cancer and poor sleep on mental health. This moderated mediation suggests that bolstering social ties and addressing sleep may have synergistic effects on improving the well-being of older cancer survivors.

## 5. Conclusions

Among middle-aged and older Chinese adults, we found that sleep quality mediate a modest portion of the association between cancer and depression, and social participation diversity moderate the impact of poor sleep on depression. These results point to sleep and social engagement as promising leverage points for reducing the depression burden in aging cancer survivors. Multidisciplinary interventions that incorporate sleep improvement and social support strategies may be beneficial to improve psychological well-being and overall quality of life in this vulnerable population.

## Author contributions

**Conceptualization:** Yingdan Bai, Yonghui Wan.

**Data curation:** Yingdan Bai.

**Formal analysis:** Yingdan Bai, Zongjing Li.

**Methodology:** Yingdan Bai, Zongjing Li, Chen Li.

**Project administration:** Qian Chen, Yonghui Wan.

**Supervision:** Qian Chen, Yonghui Wan.
